# Temperature-dependent microfluidic impedance spectroscopy for non-invasive biofluid characterization

**DOI:** 10.1063/5.0255847

**Published:** 2025-05-01

**Authors:** Tom Wade, Sohini Kar-Narayan

**Affiliations:** Department of Materials Science and Metallurgy, University of Cambridge, 27 Charles Babbage Road, Cambridge CB3 0FS, United Kingdom

## Abstract

Remote health monitoring has the potential to enable individuals to take control of their own health and well-being and to facilitate a transition toward preventative and personalized healthcare. Sweat can be sampled non-invasively and contains a wealth of information about the metabolic state of an individual, making it an excellent candidate for remote health monitoring. An accurate, rapid, and low-cost biofluid characterization technique is required to enable the widespread use of remote health monitoring. We previously introduced microfluidic impedance spectroscopy for the detection of electrolyte concentration in fluids, whereby a novel device architecture, measurement method, and analysis technique were presented for the characterization of cationic species. The purely electrical nature of this measurement technique removes the intermediate steps inherent in common rival technologies such as optical and electrochemical sensing, offering a range of advantages. In this work, we investigate the effect of temperature on microfluidic impedance spectroscopy of ionic species commonly present in biofluids. We find that the impedance spectra and concentration determination are temperature-dependent; remote health monitoring devices must be calibrated appropriately as they are likely to experience temperature fluctuations. Importantly, we demonstrate the ability of the method to measure the concentration of anionic species alongside that of cationic species, enabling the detection of chloride and lactate, which are useful biomarkers for hydration, cystic fibrosis, fatigue, sepsis, and hypoperfusion. We show that the presence of neutral species does not impair accurate determination of ionic concentration, thus, demonstrating the suitability of microfluidic impedance spectroscopy for non-invasive biofluid characterization.

## I. INTRODUCTION

Remote health monitoring (RHM) is the measurement of a patient's health outside of a typical clinical setting and includes on-demand tests that can be performed by the patient and wearable sensors.^1^ It is widely believed that RHM is vital in enabling a paradigm shift in medicine; development beyond the generalized and reactive methods currently typically used, toward methods that are individual to the patient and preventative.^2,3^ RHM enables the health of a patient to be monitored continuously and over long time periods. From this, a more detailed and accurate insight into the health of a patient can be determined, leading to more accurate and earlier diagnoses and therefore better patient outcomes. The acquisition of accurate data at suitably small time intervals and over long time periods, with minimal interference in the daily life of patients, is required for the widespread uptake of RHM.^4^

Blood testing is currently considered the gold standard for understanding the health of a patient but has many drawbacks such as lacking temporal resolution and requiring an invasive procedure. Sweat is a promising biofluid for RHM as it can be sampled non-invasively.^5,6^ It has been shown that concentrations of important biomarkers such as glucose,^7^ lactate,^8^ and ethanol^9^ in the blood and sweat are strongly correlated, giving sweat a high clinical relevance. However, there is still some conflicting evidence and associated uncertainty regarding this.^10^ This is in part due to a lack of evidence and a variety of different methodologies being followed across the literature. Table I shows the typical concentrations of some species within sweat and blood.

**TABLE I.** Chemical biomarkers in the human body and their typical concentrations in sweat and blood. Blood serum values are given.^11^ Range represents mean ± one standard deviation where relevant.

Species	Concentration in sweat (mM)	Concentration in blood (mM)
Sodium	24–56^12^	137–142^13^
Chloride	18–54^12^	96–106^14^
Urea	14–30^15^	5–7^15^
Potassium	3–7^12^	3.5–5.5^16^
Calcium	0.2–2^17^	4.3–5.3^18^
Lactate	6.5–13^12^	<2.3^19^
Ammonia	2.2–3.8^20^	8–57 × 10^−3^^21^
Glucose	0.01–0.2^17^	4.4–6.7^22^
Ethanol^a^	17.4^9,23^	14.1^23^

^a^ UK legal drink-drive limit.

To meet the clinical need to bring RHM to the masses and further investigate the relationship between sweat biomarkers and health, a suitable sensor capable of fluid characterization is required. The sensor must be capable of continuous monitoring of the small volumes of sweat available with minimal user interaction, have a long lifetime, low power requirements, good selectivity and sensitivity, and low cost. While a variety of sensor types have been developed for the analysis of biological cells,^24^ sensors capable of monitoring the concentrations of ionic species many orders of magnitude smaller than typical cells remain an unmet need. A large quantity of previous work has been conducted to develop sweat induction (for example, by iontophoresis), sampling, collection, and transport, all of which is vital for efficient, effective, and accurate sweat monitoring.^25^ There is a large demand for technology enabling remote health monitoring,^26^ and sweat sensors have been the focus of a large body of previous research,^27,28^ a range of which are summarized in Table S1 in the supplementary material.

Many sweat sensors currently in development use optical sensing.^29^ One of the most common types of optical sensing is colorimetric, in which a chemical undergoes a color change upon reaction with a specific analyte.^30^ A photograph is then taken by the user, typically on a smartphone or dedicated device, from which the color is analyzed to determine the concentration of the target analyte. However, the level of required user interaction is relatively high, and different lighting conditions and cameras require complex calibration^31,32^ and can introduce errors in the concentration determination. Fluorometric sensing is an alternative optical measurement technique, in which light, typically of a specific wavelength, is directly emitted in the presence of a target analyte. Fluorometric sensors are typically more sensitive and have a wider dynamic range than colorimetric sensors.^33^ However, accurately measuring the fluorescence intensity typically requires specialized laboratory equipment, limiting portability and increasing cost. In addition, some fluorometric sensors involve irreversible reactions, which are not suitable for RHM applications.^34^

Electrochemical detection methods are also used in the field of sweat sensing.^35,36^ This is also the most common detection method used by glucose monitors,^37^ a type of RHM device that has achieved widespread use. However, specific chemicals are required to detect each target analyte, and such chemicals often have limited storage and working lifetimes.^38^ Furthermore, for accurate measurements to be obtained, a reference electrode is required—this adds complexity, cost, and stability issues.^39^

As shown in Table S1 in the supplementary material, many of the sensors developed in the literature are only capable of measuring one analyte. In contrast, notably Koh *et al.* developed a multiplexed colorimetric sensor for measuring five analytes.^30^ In addition, a large fraction of research does not include selectivity testing, which is of high importance for RHM devices. While in some cases authors have chosen to restrict themselves to measuring physiologically relevant concentrations of target species, in other cases the limit of detection is not sufficiently low for measurements of real sweat (for example, the ascorbic acid sensor presented by Sempionatto *et al*.^40^ and the glucose sensors of Martin *et al.* and Koh *et al.*^30,41^).

Mass spectrometry can also be used to characterize sweat samples, particularly for larger molecules such as amino acids.^42^ However, although mass spectrometry has been integrated with microfluidics^43^ and miniaturization down to tabletop sizes has been achieved,^44^ the cost, size, weight and operating complexity of mass spectrometry systems remain significant barriers to their use in RHM applications.

Sweat production and the small sample volumes available continue to pose a significant problem for sweat sensing technologies.^45^ Microfluidic systems have shown promise in overcoming this issue, enabling continuous extraction of sweat during exercise, giving precise control over the fluid flow and good temporal resolution, among other benefits.^46^

In previous work, microfluidic architecture, measurement technique, and data analysis method were developed to enable the accurate characterization of an ionic fluid using purely electrical measurement methods.^47^ Interdigitated electrodes integrated within a microfluidic channel were used to measure impedance spectra. An effective capacitance (*C*_eff_) was calculated from the imaginary impedance using

Ceff=−1ω⋅Im(Z). (1)

It was found that the frequency of the inflection point of the effective capacitance as a function of frequency in log–log space was highly linearly correlated (R^2^ > 0.99) with the concentration of the cationic species in the aqueous solution within the microfluidic channel. For each cationic species, a linear relationship between the turning point frequency (TPF) and concentration was obtained and used to calculate the concentrations of solutions with unknown concentrations with a success rate of 90%. The previous study introduced the technology, focusing on cationic species; contamination from non-ionic species was not considered, and all measurements were undertaken at room temperature.

Previously ionic chloride species with different cations were investigated, but here the influence of anions is investigated by comparing the impedance spectra and TPF values of sodium chloride and sodium lactate. Anions are important within the context of RHM; for example, the concentration of chloride in sweat is related to the hydration levels and diet of an individual^48^ and is considered the gold standard for the detection of cystic fibrosis.^49^ Furthermore, lactate is also a key biomarker, which is well known to correlate with exercise intensity and fatigue.^50^ Recently, the importance of lactate as a biomarker for inflammation, cancer,^51^ cardiovascular diseases,^52^ hypoperfusion,^53^ sepsis,^54^ and other conditions has been uncovered. Additionally, sweat contains non-ionic components such as glucose.^17^ Here, the effect of glucose at a range of concentrations on the impedance spectra and TPF values measured is assessed—it is vital that non-ionic contaminants do not interfere with the characterization.

Previously, the effects of temperature on device performance were not considered. The temperature dependence (and hence required temperature compensation) of RHM sensors across the literature is often overlooked,^30,55^ despite the known temperature dependence of electrochemical^56^ detection in particular.^57^ However, in RHM applications, the temperature of the fluid and device is likely to fluctuate due to changes in the wearer's skin temperature (for example, during exercise^58^ or due to changes in climate^59^) and the ambient temperature. Therefore, importantly here the temperature dependence of the TPF–concentration relationship is investigated in detail.

Impedance spectra are well known to be temperature-dependent for a wide range of solid systems.^60,61,62^ Although impedance spectroscopy is used more rarely for the investigation of fluids than solids, it is understood that temperature is also important here due to its influence on factors such as the rate of reaction at the electrode surfaces,^63^ viscosity, conductivity, and relaxation.^64^ For example, Leys *et al.* used impedance spectroscopy to analyze the glass transition temperature, electrical conductivity, and ionic mobility of ionic liquids.^65^ In addition, Baldwin *et al.* used impedance spectroscopy measurements in aqueous solutions to measure temperature fluctuations with higher resolution than platinum resistance temperature detectors (which are typically considered the gold standard for temperature measurement), highlighting the sensitivity of impedance spectroscopy measurements to temperature.^66^

The present investigations represent an important step in the readiness of the technology for a remote health monitoring application as an on-demand or continuous sweat monitor, in addition to increasing the understanding of the device and working mechanism of the measurements.

## II. METHODS

### A. Device manufacture workflow

**FIG. 1.**
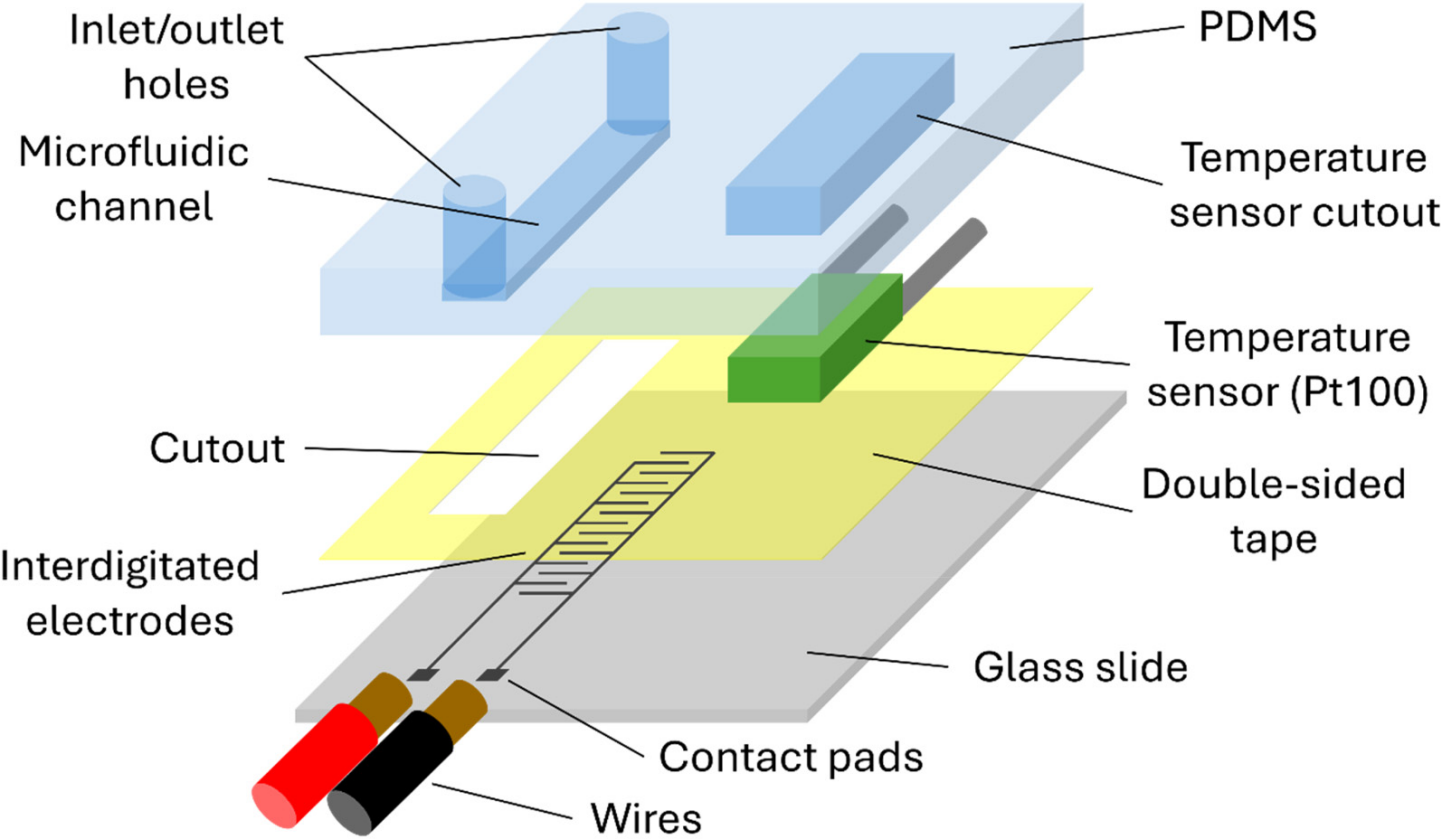
A labelled exploded diagram of the device architecture, highlighting the key components. Not to scale.

Microfluidic devices with embedded interdigitated electrodes were used to characterize aqueous solutions via impedance spectroscopy. A schematic of the device is shown in Fig. 1. Silver (JS-A221AE, NovaCentrix, Texas) interdigitated electrodes were aerosol-jet printed (Optomec AJ200, Optomec Inc., New Mexico) onto a glass slide and cured for 2 h at 200 °C. For the results presented in Sec. III A, the interdigitated regions of the electrodes were 5 mm long. For the measurements presented in Secs. III B and III C , the interdigitated regions of the electrodes were 20 mm long. The distance between the centers of neighboring interdigitated fingers was 80 *μ*m, and the electrodes were 1 mm wide. Following curing, conductive silver epoxy (8331-14G, MG Chemicals, Canada) was used to connect wires to the printed contact pads.

Polydimethylsiloxane (PDMS) (SYLGARD™ 184, Dow Inc. Michigan) was cast into 3D printed (Form 3, Formlabs, Massachusetts) molds, creating channels 5 mm longer than the interdigitated electrodes) and 1.2 mm wide, with injection/outlet holes at each end. Rectangles of the same dimensions were removed from the double-sided tape (Tesa 64621, Germany) using laser cutting (Epilog Zing 30 W, Epilog, Colorado) and were used to secure the PDMS to the glass slides such that a pair of interdigitated electrodes lay underneath each microfluidic channel. Some additional details are available in Ref. 47.

### B. Chemical solutions

Chemical solutions for testing were made up using de-ionized (DI) water filtered in-house (Purite, UK) and the relevant chemical component (Merck, Germany). The target solution concentration values are quoted for simplicity in the text, but the actual (measured) concentrations were used for plotting and linear regression throughout. The target and actual (measured) concentrations of the single-species solutions used in the room-temperature measurements presented in Sec. III A are shown in Table S2 in the supplementary material. For the NaCl solutions, the average relative error magnitude was 0.030%, and the largest error magnitude was 0.084%. For the sodium lactate (NaLac) solutions, the average relative error magnitude was 0.021%, and the largest error was 0.034%.

The target and actual (measured) concentrations of the two-species NaCl and glucose solutions used in the measurements presented in Sec. III B are shown in Table S3 in the supplementary material. The average relative NaCl error magnitude was 0.043%, and the largest absolute error was 0.22%. The average relative glucose error magnitude (excluding solutions without glucose) was 0.066%, and the largest error magnitude was 0.174%.

The target and actual (measured) concentrations of the single-species ionic chloride solutions used in the temperature-dependent measurements presented in Sec. III C are shown in Table S4 in the supplementary material. Over all four species, the average relative error magnitude was 0.013% and the largest error magnitude was 0.0271%.

### C. Room temperature experimental procedure

Measurements of a range of aqueous ionic solutions were carried out using microfluidic impedance spectroscopy at room temperature. Syringes were used to inject the test solutions into the microfluidic channels. Fluid was removed by wicking with paper towels. The channels were flushed with the solution to be tested next. The solution was removed by wicking and re-injected, then an impedance spectrum was measured (see Sec. II D)—this was repeated thrice. The solutions were injected in order of increasing concentration (to minimize cross-contamination), with the highest two concentrations switched to separate concentration-dependence from time-dependence. After each injection, the channel was visually inspected for bubbles, and the solution was removed and re-injected if they were observed.

Three channels were injected and measured simultaneously for each repeat of each solution. Anomalies (e.g., produced by poor electrical connections or bubbles in the channels) were removed, and the presented results represent an average and one standard deviation from the remaining data unless otherwise specified. Some additional experimental details are described in Ref. 47.

### D. Impedance spectroscopy measurements

Impedance measurements were acquired using a Sciospec ISX-3v2 impedance analyzer (Sciospec Scientific Instruments GmbH, Germany). The samples were connected via a SlideChipAdapter (Sciospec Scientific Instruments GmbH). Voltage-controlled mode was used, with an excitation voltage amplitude of 250 mV. A measurement range of ±100 *μ*A was used, as recommended by the manufacturer for impedance values of <1 kΩ. Measurements were made in 2048 logarithmically spaced steps between 10 kHz and 25 MHz inclusive. A precision setting (described by the manufacturer as being “directly correlated to the relative bandwidth of the measurement”) of 1 was used.

Before each experiment, the impedance analyzer was calibrated using an open circuit, a closed circuit, and a 330 Ω resistor, to remove the effect of parasitic capacitance in the system. Any effects of contact resistance, parasitic capacitance, and coupling capacitance have been mitigated by maintaining the length and position of wires connecting the impedance analyzer to the electrodes throughout each experiment and using the same electrodes to test each solution, eliminating sample-to-sample variation.

### E. Temperature-dependent measurements

**FIG. 2.**
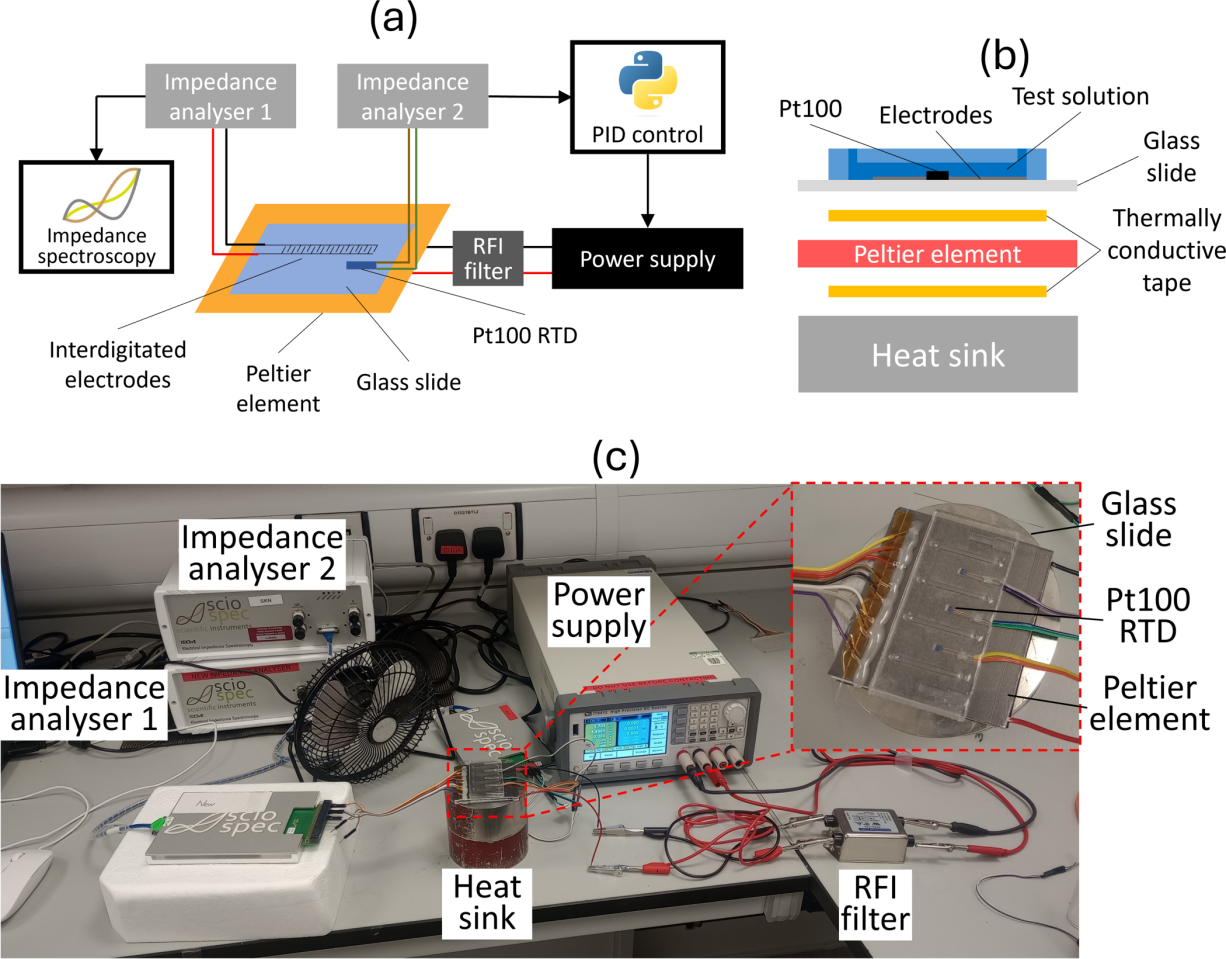
(a) Labelled schematic of the experimental setup used to acquire impedance spectroscopy measurements at a range of temperatures. (b) Labelled schematic of a cross section of the heat sink, Peltier element, and device stack. (c) Photograph of the experimental setup. As described in the text, the setup includes two impedance analyzers, two computers (not shown), a sample containing interdigitated electrodes, Pt100 resistance temperature detectors [one of each shown in (a) for clarity], a power supply, a Peltier module, and a radio frequency interference (RFI) filter. (a) and (b) Not to scale.

A schematic and a photograph of the temperature-dependent experimental setup are shown in Fig. 2. During temperature-dependent measurements, the impedance spectra were measured as described in Sec. II D. The impedance spectra were measured continuously, with an acquisition time of approximately 5 s per channel, measuring three channels in turn. The temperature of the sample was controlled using a 17.2 W, 62 × 62 mm Peltier module (European Thermodynamics, UK), with power for cooling and heating provided by an ITECH IT6412 DC power supply (ITECH, Taiwan). A 5 A, 250 V single phase chassis mount radio frequency interference (RFI) filter (Roxburgh EMC, UK) was used in series with the power supply and Peltier module to reduce electrical noise. A metal cylinder was used as a heat sink for the Peltier element. A double-sided self-adhesive graphite thermal interface sheet (RS PRO, UK) with a thickness of 0.045 mm was used on both sides of the Peltier module to ensure good thermal contact with the device and heat sink.

The temperature of the sample was measured using three Class A Pt100 resistance temperature detectors (RTDs) (RS PRO, UK), which were situated in cutouts in the PDMS (see Fig. 1). The resistance of these was measured continuously by a second Sciospec ISX-3v2 impedance analyzer, at a measurement frequency of 1 kHz. Typically three Pt100 RTDs were used, spaced across the sample, and the temperature was calculated from the average of the three measurements. The linear relationship between Pt100 resistance and temperature is well known.^67^ Contributions of additional resistances (e.g., wires, solder) were removed using calibration at room temperature.

The voltage supplied to the Peltier element was controlled via a proportional-integral-derivative (PID) controller written in Python. The resistance value was calculated from the absolute and phase values, and the temperature was calculated using the known resistance–temperature relationship of Pt100 RTDs after applying the room-temperature calibration. This was carried out for each of the Pt100s, and the temperature was compared to the temperature setpoint by the PID controller, which calculated the required voltage for the Peltier element. The voltage of the power supply was updated by the PID controller typically every 100–150 ms. The temperature was ramped at 2.5 °C min^−1^ to minimize thermal lag; a full temperature profile is shown in Fig. S1 in the supplementary material. The impedance analyzers were started simultaneously, to enable the temperature data and impedance spectra to be matched during data analysis. Three channels were measured per temperature sweep, each containing the same solution. The temperature profile was repeated for different solutions. One temperature sweep was carried out per test solution, with errors determined by the standard deviations between the three measured channels, and multiple (typically two) spectra measured per degree Celsius. The channels were flushed and re-used between runs of different solutions as described in Sec. II C.

All experiments were conducted within the controlled laboratory environment, and therefore, the humidity was relatively constant. Humidity is not expected to influence the impedance spectroscopy measurements in the same manner as temperature (except via affecting evaporation rates over long time periods), as both electrodes are already in direct contact with the aqueous fluid within the microfluidic channel.

### F. Turning point frequency analysis

The frequency of the turning point of the effective capacitance–frequency plot in log–log space (turning point frequency, TPF) was determined. The effective capacitance was calculated using Eq. (1). The effective capacitance-frequency curve was smoothed and differentiated twice (in log-log space), and the point of inflection was identified at the point where the second differential was equal to zero. This point was calculated to high accuracy using linear interpolation between the two nearest datapoints. This was the TPF value for the impedance spectrum. This was calculated for each measurement for room-temperature or temperature-dependent measurements. Some additional details are available in Ref. 47.

### G. Nyquist analysis

Based on the shape of the Nyquist plots [see Fig. 8(a)], the Randles circuit was identified as the equivalent circuit for the proposed architecture.^68^ The Nyquist plot has two distinct regions: an arc and a linear region. A semicircle and a straight line were fitted to these regions, respectively, with the center of the semicircle fixed at y = 0. Values for the components of the equivalent circuit were calculated as shown in Fig. S6 in the supplementary material.

## III. RESULTS AND DISCUSSION

### A. Anion comparison: Sodium chloride vs sodium lactate

**FIG. 3.**
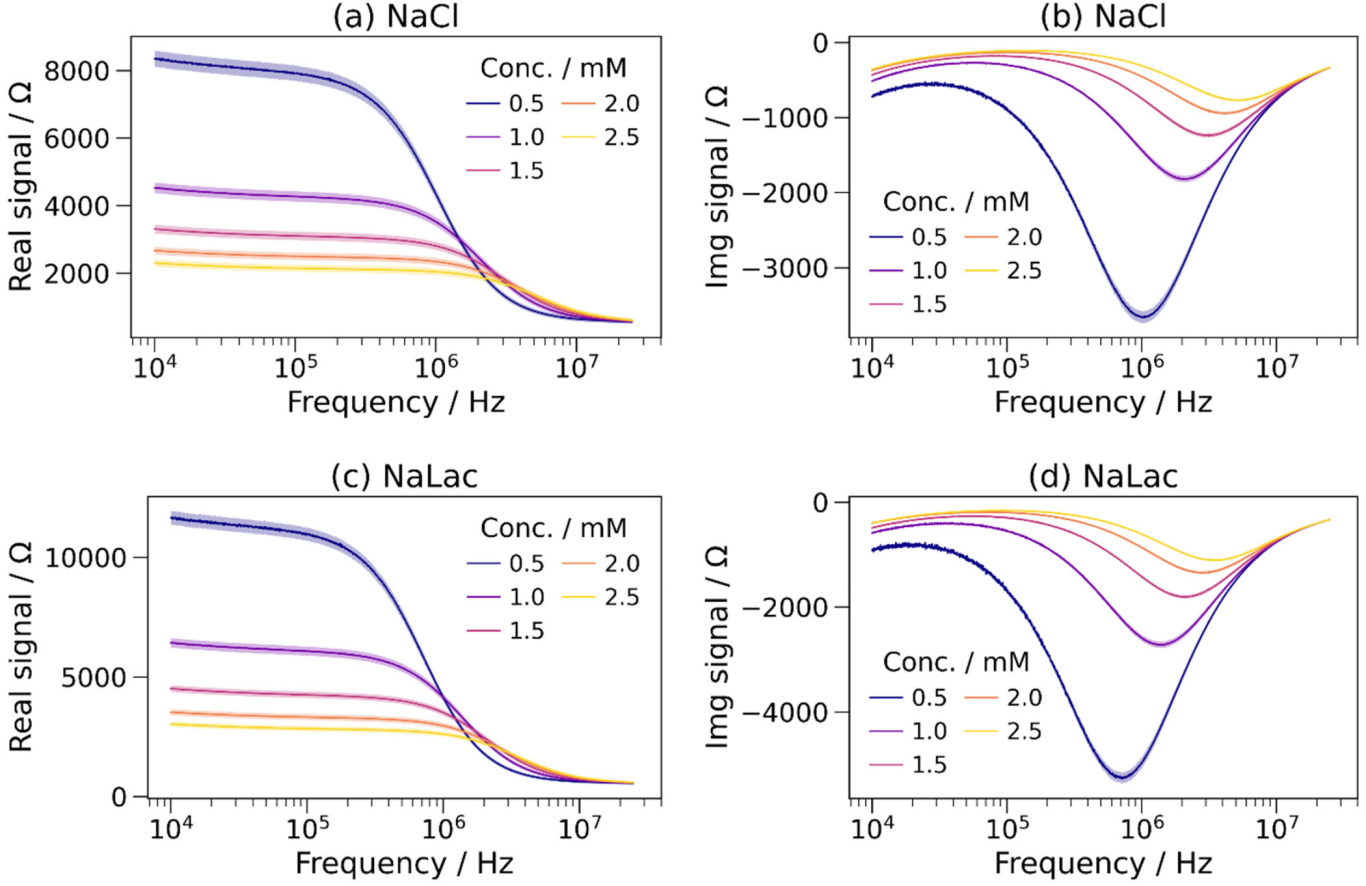
(a) Real and (b) imaginary spectra of NaCl solutions; (c) real and (d) imaginary spectra of sodium lactate solutions, with concentrations from solutions with concentrations from 0.5 to 2.5 mM. Shaded regions represent one standard deviation over three measurements of each of three channels.

Sodium chloride (NaCl) and sodium lactate (NaLac) solutions with a range of concentrations were measured using the method described in Sec. II C. Exact concentrations of these solutions are shown in Table S2 in the supplementary material. The real and imaginary impedance spectra measured using the described devices are shown in Fig. 3.

At most measured frequencies, the absolute values of the real and imaginary impedances decreased with increasing ionic concentration. At low frequencies (10 kHz), the real impedance was large (8.5 kΩ for 0.5 mM NaCl) but decreased significantly from 1 to 10 MHz (to 610 Ω at 25 MHz for 0.5 mM NaCl). The magnitudes of the imaginary impedance spectra were small at the lowest and highest tested frequencies, with a minimum between. The minimum in the imaginary spectra increased in frequency with increasing concentration—for example, it increased from 1 to 5 MHz when NaCl concentration was increased from 0.5 to 2.5 mM.

**FIG. 4.**
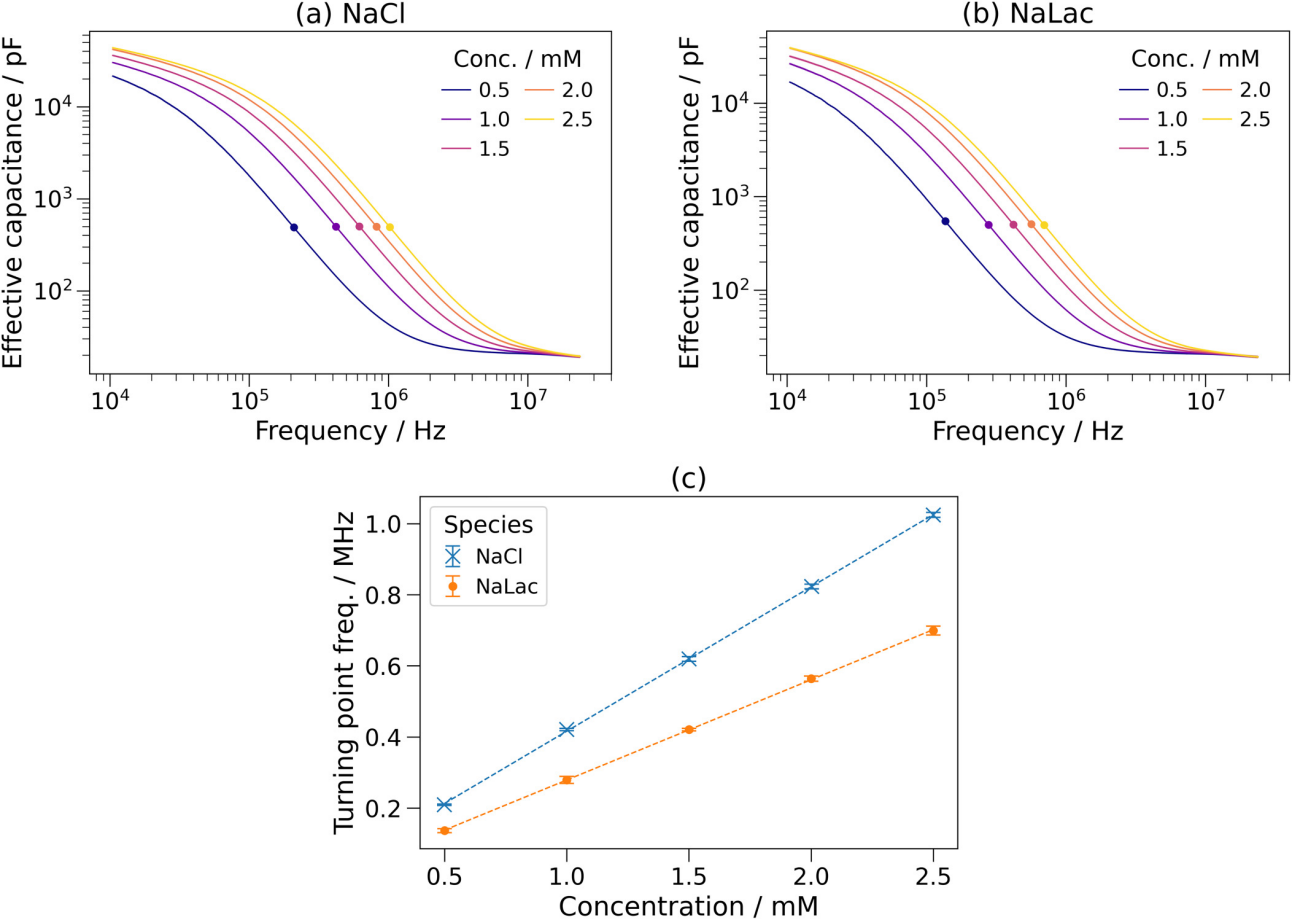
The effective capacitance as a function of frequency for (a) sodium chloride (NaCl) and (b) sodium lactate (NaLac) solutions with concentrations from 0.5 to 2.5 mM. Turning points are marked with dots. One standard deviation over three measurements of each of three channels was similar to the linewidth so is not shown. (c) Turning point frequency–concentration relationship for sodium chloride and sodium lactate. Error bars show one standard deviation over three measurements of each of three channels, and dashed lines show linear fits to each dataset.

The effective capacitance was calculated using Eq. (1). Figures 4(a) and 4(b) show the effective capacitance as a function of frequency for the NaCl and NaLac solutions. As previously observed,^47^ the capacitance was large at low (∼10 kHz) frequencies and orders of magnitude smaller at MHz frequencies, with an inflection point between.

The turning point frequency (TPF) of each solution is shown in Fig. 4(c). The TPF–concentration relationship is highly linear, with R^2^ > 0.9999 for both NaCl and NaLac. However, notably the gradients of the TPF–concentration linear fits were different; 0.406 and 0.282 MHz mM^−1^ for NaCl and NaLac, respectively. While the previous work focused on the relationship between cations and the TPF, this shows that the TPF is also influenced by anions.

As discussed in Sec. I and in Ref. 47, the shape of the effective capacitance–frequency curve is believed to be produced by Stern layer formation at low frequencies, which cannot occur at high frequencies due to the time taken for ionic migration. This is depicted in Fig. S3 in the supplementary material. Therefore, solutions containing ions that can move at a higher velocity (i.e., have a higher ionic mobility) have a higher turning point frequency, as the Stern layer formation time is lower.

Ionic mobility (*μ*) is dependent on the Stokes radius (also called hydrodynamic radius) (*r*) and can be calculated using the following equation:

$$\mu =\frac{ez}{6\pi \eta r},$$ (2)

where *e* is the charge of an electron, *z* is the ionic charge, and *η* is the viscosity of the fluid. The Stokes radii and ionic mobilities of the chloride (Cl^−^) and lactate (Lac^−^) anions are shown in Table II. Due to its larger size, the ionic mobility of the lactate anion is lower than that of the chloride anion. This is in agreement with the TPF of a NaLac solution being lower than that of a NaCl solution with the same concentration, as observed in Fig. 4(c), supporting the ionic migration-mediated Stern layer formation theory. This theory will be investigated further using measurements at a range of temperatures in Sec. III C.

**TABLE II.** Stokes radii and ionic mobilities of chloride and lactate anions in water at 20 °C.

Ion	Stokes radius (× 10^−10^ m)	Ionic mobility (× 10^−8^ m^2^ s^−1^ V^−1^)
Cl^−^	1.8^69^	4.68
Lac^−^	2.2^70^	3.85

It has been shown that anions, in addition to cations, affect the measured impedance spectra and the TPF values. This supports the previously reported theory, as the anionic and cationic species form Stern layers at the anode and cathode, respectively, and so are expected to contribute to the TPF independently, as shown in Fig. S3 in the supplementary material. This had not previously been shown and must be considered when using microfluidic impedance spectroscopy to analyze ionic solutions, particularly for complex solutions such as sweat.

### B. Influence of non-ionic contaminants

Sweat is a complex biofluid containing a variety of ionic and non-ionic species. Glucose is an example of a non-ionic chemical present in sweat. The concentration of glucose in sweat varies over time and from person-to-person due to factors such as diet and exercise. Therefore, it is important that glucose and other non-ionic molecules do not influence the measured impedance spectra in such a way that the turning point frequency is affected, as this would give rise to incorrect concentration measurements of the ionic species.

**FIG. 5.**
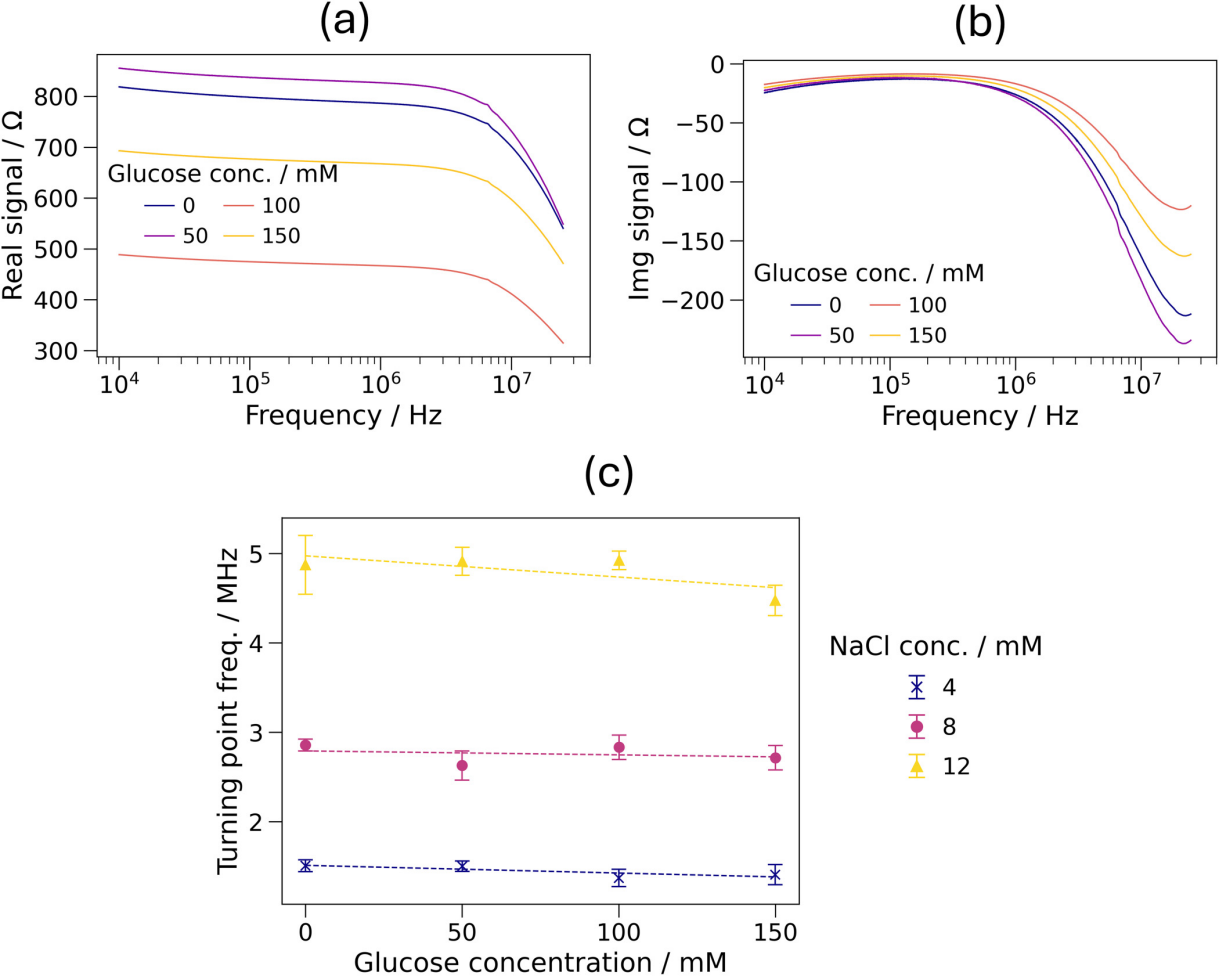
(a) Real and (b) imaginary impedance spectra of 8 mM NaCl solutions containing 0 to 150 mM glucose. Shaded regions representing one standard deviation over three measurements of each of three channels were excluded for clarity due to significant overlap. (c) Turning point frequencies of 4, 8, and 12 mM NaCl solutions containing 0 to 150 mM glucose. Dashed lines represent linear fits to the data. Error bars represent one standard deviation over three measurements of each of three channels.

To investigate this, NaCl solutions with glucose concentrations of up to 150 mM—far higher than those found in sweat—have been tested. The exact concentrations of the solutions used are shown in Table S3 in the supplementary material. Figures 5(a) and 5(b) show the real and imaginary impedance spectra of solutions containing 8 mM NaCl and 0–150 mM glucose. No relationship was observed between the glucose concentration and the impedance spectra. Figure 5(c) shows the turning point frequencies of solutions containing 4–12 mM NaCl and 0–150 mM glucose. The gradients of the linear fits were (−2.2 ± 1.5) kHz mM^−1^. This suggests that it is possible that the concentration of glucose may have a small effect on the TPF value. The typical concentration range of glucose in sweat is 0.01–0.2 mM.^17^ At the upper limit of this range, this would cause a change in the TPF value of 0.44 kHz. Using the TPF–concentration gradient obtained for NaCl in Sec. III A (721 kHz mM^−1^), this would give an error in NaCl concentration determination of 0.6 *μ*M. This is less than 0.01% of the typical concentrations of sodium and chloride in sweat.^17^ Therefore, it has been shown that glucose does not pose a significant, if any, contamination risk to the proposed sensing architecture.

### C. Temperature dependence

As described in Sec. I, the temperature of the device (and hence the sweat in the microfluidic channel) is likely to change during use, and therefore, the effects of temperature on the impedance spectra and turning point frequency must be investigated. Furthermore, if the effect of temperature is dependent on the type of ion and/or its concentration, then there exists the possibility of using temperature-dependent effects to aid selectivity.

The effect of temperature was investigated using the experimental setup and procedure detailed in Sec. II E. Exact concentrations of the solutions used are shown in Table S4 in the supplementary material.

#### 1. Turning point frequency analysis

**FIG. 6.**
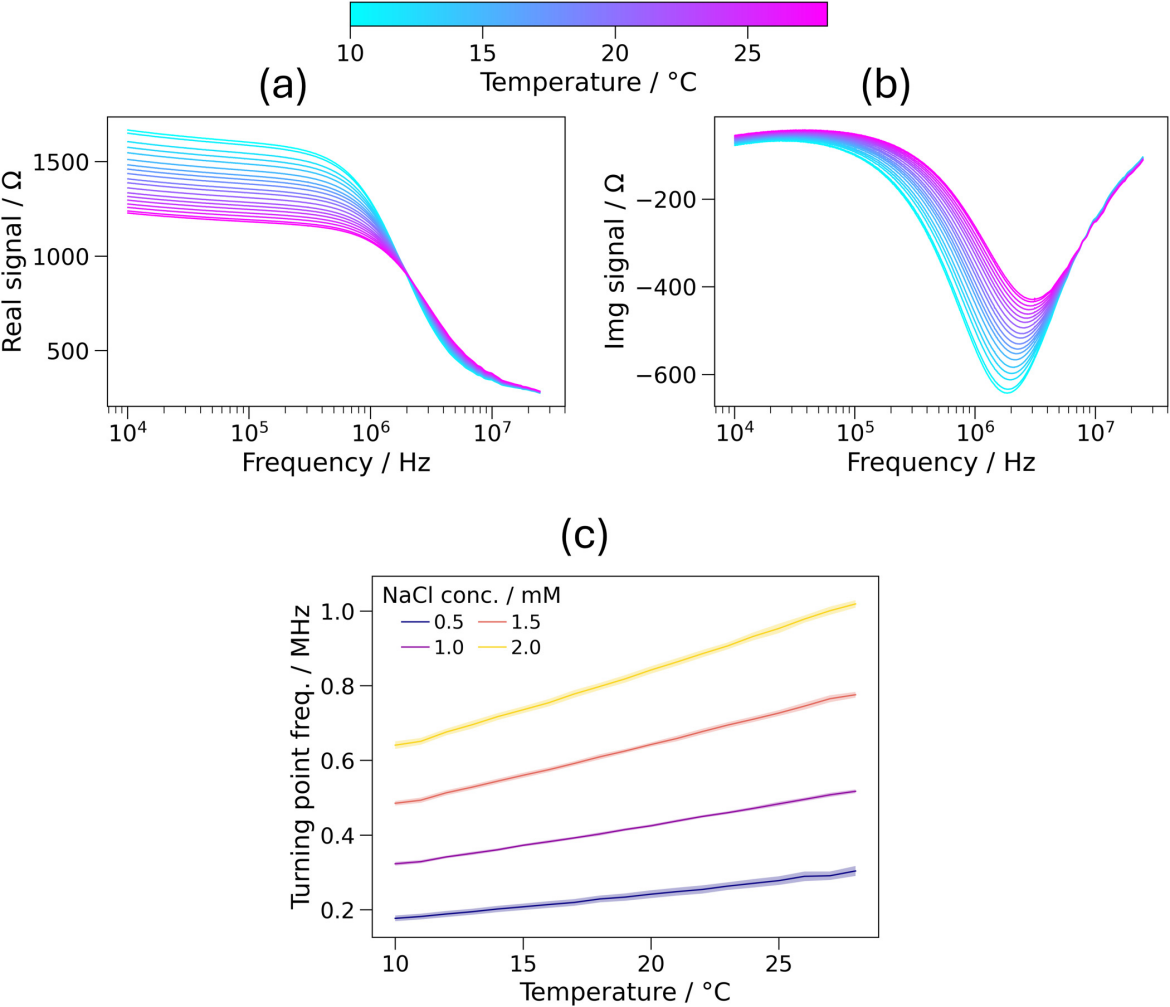
(a) Real and (b) imaginary impedance spectra of a 1 mM NaCl solution at temperatures from 10 to 28 °C. One line per degree Celsius is shown, measured from a single channel. (c) Turning point frequencies of NaCl solutions from 0.5 to 2 mM at temperatures from 10 to 28 °C. Shaded regions represent one standard deviation over three channels.

Figures 6(a) and 6(b) show the real and imaginary impedance spectra, respectively, of a 1 mM NaCl solution measured at temperatures from 10 to 28 °C. These measurements were obtained with increasing temperature, and analogous results were obtained with decreasing temperature—see Fig. S4 in the supplementary material. It is, therefore, clear that the real and imaginary components of the impedance spectra were influenced by temperature, as the results were shown to be independent of time (indicating negligible sensor drift and evaporation). As the temperature was increased, at most frequencies the magnitude of the real and imaginary components decreased. The minimum of the imaginary spectrum also moved to higher frequencies, from 1.8 MHz at 10 °C to 3.1 MHz at 28 °C.

As shown in Fig. 6(c) for NaCl solutions with solutions of 0.5–2 mM, the turning point frequency was also found to be dependent on temperature. For example, the TPF of 1 mM NaCl increased from (323 + 5) to (517 + 5) kHz (a 60% increase) from 10 to 28 °C (a gradient of 11.0 kHz °C^−1^). The relationship between the TPF and temperature was found to be linear, with an average R^2^ value for the four lines shown in Fig. 6(c) of 0.999. If not calibrated for, using the gradient of the TPF–concentration relationship found in Sec. III A, for a 1 mM solution, this would give an error in concentration determination of 27 *μ*M °C^−1^, which could be significant given the relatively wide temperature range that could be experienced by the device during typical use in remote health monitoring applications.

Based on the previously presented theory, the TPF is dependent on the velocity of the movement of the ions and the ionic concentration. The ionic mobility (*μ*) is dependent on the fluid viscosity (*η*), as shown in Eq. (2). In turn, *η* is dependent on the temperature. The viscosity of liquids as a function of temperature *T* is given by the Vogel–Fulcher–Tammann (VFT) equation,

$$\eta (T)=e^{A+\frac{B}{T+C}}\;,$$ (3)

where for water at 273 K < *T* < 373 K, the constants are empirically found to be *A* = −3.7188, *B* = 578.92, and *C* = −137.55.^71^ Table III shows the calculated viscosity of water and the corresponding ionic mobility of sodium cations (Na^+^) at 10 and 28 °C, calculated using the Stokes radius r = 1.84 Å.^69^ The increase in ionic mobility of Na^+^ from 10 to 28 °C is 55%. See Fig. S5 in the supplementary material for plots of the viscosity of water and the ionic mobility of chloride anions as a function of temperature.

**TABLE III.** The viscosity of water [calculated using the Vogel-–Fulcher–Tammann equation, Eq. (3)] and ionic mobility of sodium cations at 10 and 28 °C.

Temperature, *T* (°C)	Temperature, *T* (K)	Water viscosity, *η*(*T*) (mPa s)	Na^+^ ionic mobility, *μ*(Na^+^, *T*) (× 10^−8^ m^2^ s^−1^ V^−1^)
10	283	1.30	3.55
28	301	0.84	5.51

The capacitance is also influenced by the dielectric constant (*ε*), which is also a function of temperature,

$$\epsilon (T)=aT^{2}+bT+c,$$ (4)

where, for pure water, empirically determined coefficients (calculated from data in Ref. 72 for temperatures given in Celsius) *a* = 7.36 × 10^−4^, *b* = −0.401, and *c* = 88.18. Therefore, *ε*(10 °C) = 84.2 and *ε*(28 °C) = 77.5, a decrease of 8.0%. This is a much smaller change than that in ionic mobility and will become insignificant above relatively small fractions of Stern layer formation, and hence, it is not believed to affect the TPF. See Figure S5 in the supplementary material for a plot of the relative dielectric constant of water as a function of temperature.

**FIG. 7.**
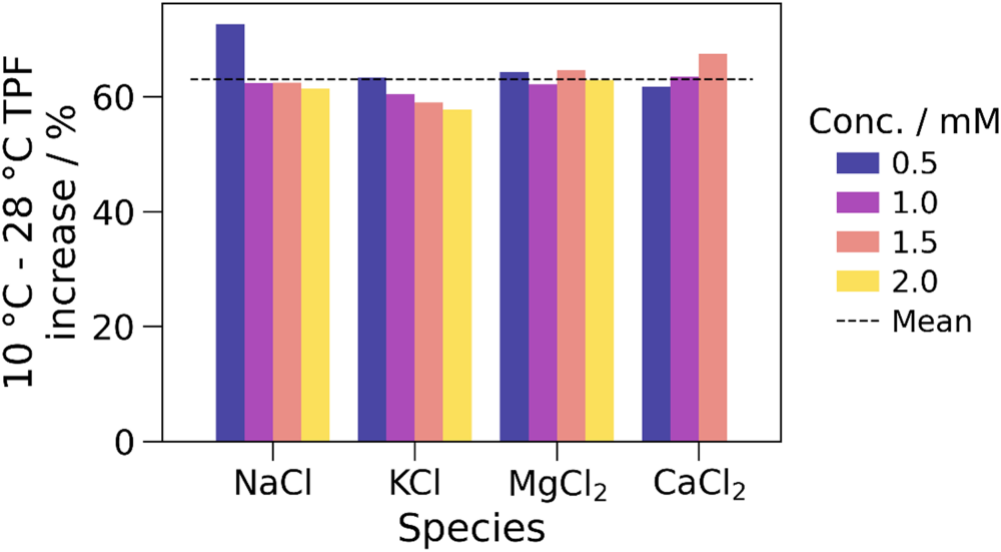
The percentage increase in turning point frequency (TPF) between 10 and 28 °C for solutions of NaCl, KCl, CaCl_2_, and MgCl_2_ with concentrations from 0.5 to 2 mM. The average of all values is shown by the dashed line. The 2 mM CaCl_2_ solution is omitted as it was anomalous.

As shown in Fig. 7, the relative increase in TPF with increasing temperature (specifically from 10 to 28 °C) was found to be independent of the ionic species and its concentration. Across the solutions shown in Fig. 7 (15 solutions, as 2 mM CaCl_2_ was anomalous and therefore removed), the percentage increase was found to be (63 ± 4)%. The relative error of 5.6% highlights that the fractional TPF increase is independent of the ionic species and concentration. Although (63 ± 4)% does not include the calculated 55% change in ionic mobility, it is noted that due to the exponential nature of the VFT equation, a small inaccuracy in the temperature readings could be the cause of this—for example, a 59% change in ionic mobility (within one standard deviation of the average relative TPF increase) would be expected from 9.5 to 28.5 °C. Therefore, the increase in TPF with increasing temperature is explained by the increase in ionic mobility caused by the decrease in viscosity. The significant impact of such small changes in temperature highlights a crucial advantage of microfluidics over bulk measurements for this application, to minimize temperature gradients within the fluid.

Although the ionic mobility is non-linear as a function of temperature, over a small temperature range it can be approximated as linear. The expected ionic mobility values from 10 to 28 °C have R^2^ = 0.999 (see Fig. S5 in the supplementary material), so it is unsurprising that this small non-linearity is not observable in the experimental data over this temperature range.

Therefore, it has been shown that the effect of temperature on the turning point frequency of a solution is dependent on only the properties of the solvent (water) and not on the properties of the ions. Hence, measurements at different temperatures cannot provide specificity to the sensors, as all solutions are affected in the same way relative to their TPF values at a specific temperature.

However, clearly it is vital that the effects of temperature are calibrated against, as significant changes (63%) in the TPF have been observed across a relatively moderate temperature range that could feasibly occur during service due to changes in ambient, sweat, and skin temperature. As shown, across a suitably small temperature range, the calibration could be approximated as linear, but over a wider temperature range non-linearities must be accounted for to ensure high accuracy. Equations (3) and (4) apply outside of the tested temperature range, and so the conclusions here are expected to hold, aside from the aforementioned non-linearity. However, a change in the coordination number of ions with respect to temperature is expected to affect the Stokes radius and, therefore, the TPF—this limits the applicability of this calibration method, and for accurate calibration, this must be accounted for where relevant. This was not observed for the tested conditions in this study. If no coordination number changes are expected for the ions in solution across the relevant temperature range, then Fig. 7 shows that the same temperature calibration can be applied to all solutions, irrespective of their ionic composition.

#### 2. Nyquist analysis

**FIG. 8.**
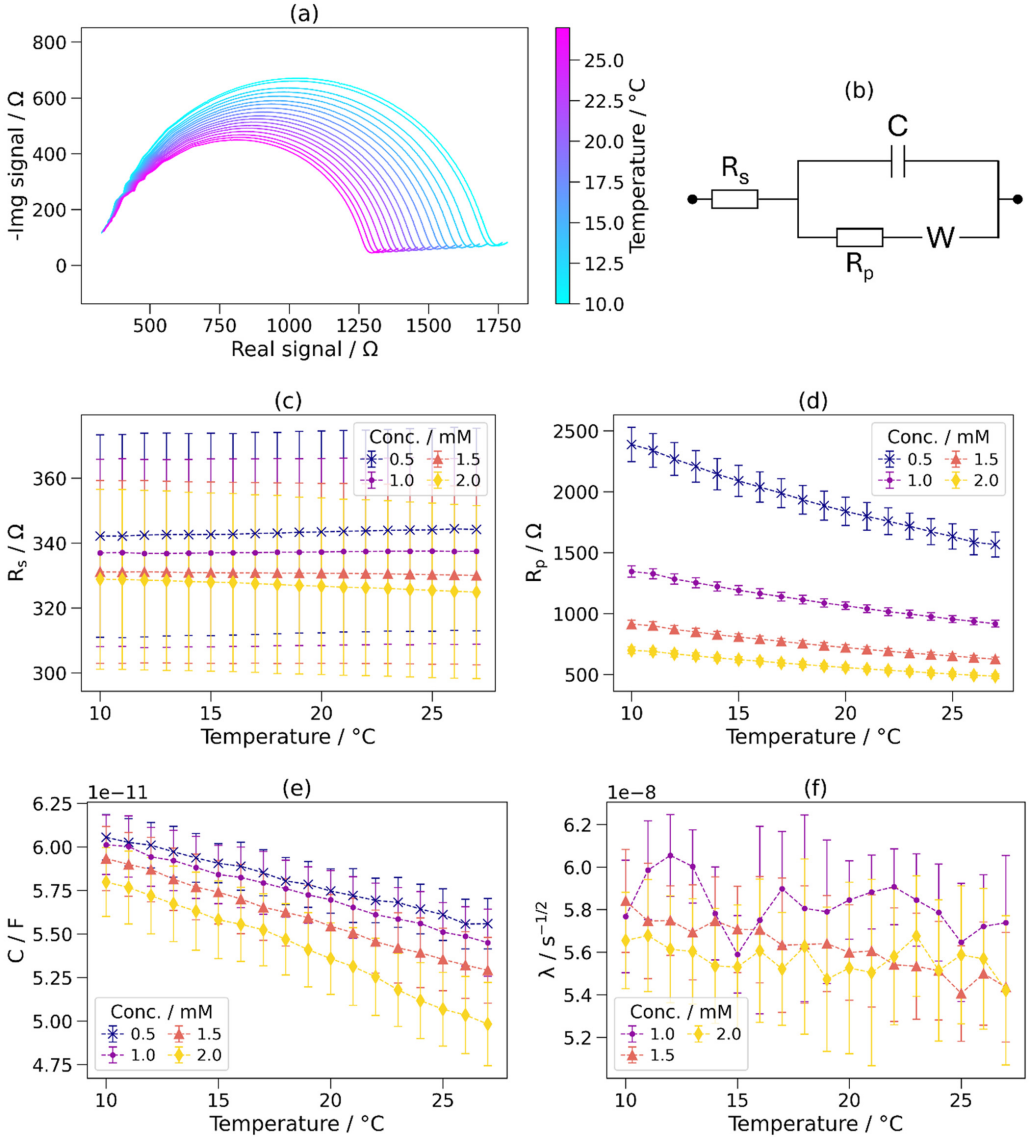
Nyquist analysis of NaCl solutions at a range of temperatures. (a) The Randles equivalent circuit used. (b) Nyquist plots of 1 mM NaCl from 10 to 27 °C, measured using a single channel. (c)–(f) Series resistance (*R_s_*), parallel resistance (*R_p_*), capacitance (C), and Warburg coefficient (*λ*) for 0.5–2 mM NaCl solutions from 10 to 27 °C. 0.5 mM NaCl is not shown in (f) due to anomalous results.

Figure 8(a) shows Nyquist plots and analysis of NaCl solutions at a range of temperatures. The Nyquist plots have a strong temperature dependence—with increasing temperature, the radius of the arc decreases. For further analysis, the Randles equivalent circuit shown in Fig. 8(b) (identified based on the shape of the Nyquist plots) was fitted as described in Sec. II G. Analogous results were also obtained for KCl, MgCl_2_, and CaCl_2_ solutions—p values and gradient (m) values given are an average over all tested species and concentrations, except for *λ*, for which 0.5 mM solutions of monovalent species were excluded as anomalous values were obtained due to their small linear region.

The series resistance (*R_s_*) was observed to be independent of temperature. For each solution, the resistance either increased or decreased with respect to temperature, giving a low average Pearson correlation value of p = 9 × 10^−4^. However, the gradient (m) was (−0.16 ± 0.21) Ω °C^−1^, and therefore, no significant relationship between the series resistance and temperature was found, and the series resistance is interpreted as not including the electrical resistance of the solution to a significant extent.

The parallel resistance (*R_p_*) decreased with increasing temperature for all tested solutions [p < 10^−3^, m = (−18 ± 13) Ω °C^−1^]. The absolute decrease of *R_p_* was greater at lower solution concentrations—for example, it was −49 and −12.7 Ω °C^−1^ for 0.5 and 2 mM NaCl solutions, respectively. However, the relative decrease in *R_p_* was found to be independent of the ionic species and concentration at (−2.2 ± 0.2)% °C^−1^ relative to the parallel resistance at 20 °C. This is for the same reasons as previously described for the TPF–temperature relationship. The parallel resistance is interpreted as being the resistance across the fluid sample, which decreases with increasing temperature and increasing ionic concentration due to the increase in conductivity of the liquid.

The capacitance of the solutions decreased with increasing temperature, as shown in Fig. 8(e) [p < 10^−3^, m = (−0.43 ± 0.11) pF °C^−1^]. Relative to the capacitance at 20 °C, the capacitance decreased by (0.84 ± 0.26)% °C^−1^, which is greater than the value of 0.46% °C^−1^ predicted by Eq. (4). The capacitance decreased by a greater amount for solutions with higher concentrations; for example, the capacitance–temperature gradients of 0.5 and 2 mM NaCl solutions were −0.30 and −0.49 pF °C^−1^, respectively.

No relationship was found between the Warburg coefficient (*λ*) and temperature. The average Pearson correlation coefficient was p = 0.07. The *λ*-temperature gradient was (0.5 ± 2) × 10^−10^ s^−1/2^ °C^−1^. It is expected that *λ* increases with temperature due to an increase in the ion migration rate. The expected decrease in ionic mobility relative to its value at 20 °C [calculated using Eqs. (2) and (3)] is 2.4% °C^−1^. The *λ* value is dependent on the previously determined parameters (see Fig. S6 in the supplementary material), which can cause errors to propagate, which may mask this change.

Nyquist analysis has determined that the parallel resistance (*R_p_*) and capacitance (*C*) both exhibit trends with respect to temperature in addition to solution concentration, likely to be predominantly due to changes in the conductivity and dielectric constant, respectively. The series resistance (*R_s_*) and Warburg coefficient (*λ*) were not found to be dependent on temperature.

## IV. CONCLUSIONS

The previously developed microfluidic architecture and measurement technique has been further investigated, as it offers a variety of benefits relative to more common technologies. It has been shown that, in addition to cations, anions also affect the impedance spectra and the effective capacitance–frequency turning point frequency (TPF). Therefore, the concentration of anionic species can also be determined using the presented architecture, and this must also be considered during data interpretation. For example, as suggested in the previous work, the difference between monovalent and divalent ionic chloride species is not only due to the difference in ionic mobility of the cation but also the difference in anion concentration. Anion concentration determination enables the measurement of species such as chloride and lactate, which are important in remote health monitoring applications. Furthermore, it has been shown that the presence and concentration of glucose, a non-ionic species, do not significantly influence the determination of ionic concentrations. However, temperature has been proven to significantly influence the measured impedance spectra and TPF values, mainly due to its effect on the conductivity and dielectric constant of the fluid, as determined by Nyquist analysis. Therefore, the temperature must be measured and calibration used to achieve accurate and reliable ionic fluid characterization using microfluidic impedance spectroscopy. The observed results have supported the previously proposed ionic mobility-limited Stern layer formation theory that explains the form of the effective capacitance as a function of frequency, and the relationship between the TPF and ionic species, ionic concentration, temperature, and other parameters. However, some areas of further investigation and development exist, in particular, before the technology can be used within a wearable sensor. For example, the selectivity, effect of movement, electrode degradation, and the power consumption of measurement acquisition, processing, and transfer must be addressed. Despite this, here important steps have been made toward the utilization of microfluidic impedance spectroscopy for remote health monitoring applications.

## SUPPLEMENTARY MATERIAL

See the supplementary material for a table summarizing the previous literature, exact solution concentrations, additional data characterizing the temperature-dependent experimental setup, the temperature-dependence of water properties, and the method used to determine Randles equivalent circuit element values from Nyquist plots.

## Data Availability

The data that support the findings of this study are openly available in Apollo University of Cambridge Repository at https://doi.org/10.17863/CAM.115876, Ref. 73.

## References

[1] L. P. Malasinghe, N. Ramzan, and K. Dahal, “Remote patient monitoring: A comprehensive study,” J. Ambient Intell. Hum. Comput. 10, 57–76 (2019). 10.1007/s12652-017-0598-x

[2] J. Kulkova, I. Kulkov, R. Rohrbeck, S. Lu, A. Khwaja, H. Karjaluoto, and J. Mero, “Medicine of the future: How and who is going to treat us?,” Futures 146, 103097 (2023). 10.1016/j.futures.2023.103097

[3] O. Golubnitschaja, V. Costigliola and EPMA, “General report & recommendations in predictive, preventive and personalised medicine 2012: White paper of the European Association for Predictive, Preventive and Personalised Medicine,” EPMA J. 3, 14 (2012). 10.1186/1878-5085-3-14 23116135 PMC3485619

[4] S. Majumder and M. J. Deen, “Smartphone sensors for health monitoring and diagnosis,” Sensors 19(9), 2164 (2019). 10.3390/s19092164 31075985 PMC6539461

[5] J. Xu, Y. Fang, and J. Chen, “Wearable biosensors for non-invasive sweat diagnostics,” Biosensors 11(8), 245 (2021). 10.3390/bios11080245 34436047 PMC8391966

[6] S. Jadoon, S. Karim, M. R. Akram, A. Kalsoom Khan, M. A. Zia, A. R. Siddiqi, and G. Murtaza, “Recent developments in sweat analysis and Its applications,” Int. J. Anal. Chem. 2015, 164974. 10.1155/2015/164974 25838824 PMC4369929

[7] J. R. Sempionatto, J.-M. Moon, and J. Wang, “Touch-Based fingertip blood-free reliable glucose monitoring: Personalized data processing for predicting blood glucose concentrations,” ACS Sens. 6(5), 1875–1883 (2021). 10.1021/acssensors.1c00139 33872007

[8] D. A. Sakharov, M. U. Shkurnikov, M. Yu. Vagin, E. I. Yashina, A. A. Karyakin, and A. G. Tonevitsky, “Relationship between lactate concentrations in active muscle sweat and whole blood,” Bull. Exp. Biol. Med. 150, 83–85 (2010). 10.1007/s10517-010-1075-0 21161059

[9] M. J. Buono, “Sweat ethanol concentrations are highly correlated with co-existing blood values in humans,” Exp. Physiol. 84(2), 401–404 (1999). 10.1017/s0958067099017984 10226180

[10] L. Klous, C. J. de Ruiter, S. Scherrer, N. Gerrett, and H. A. M. Daanen, “The (in)dependency of blood and sweat sodium, chloride, potassium, ammonia, lactate and glucose concentrations during submaximal exercise,” Eur. J. Appl. Physiol. 121, 803–816 (2021). 10.1007/s00421-020-04562-8 33355715 PMC7892530

[11] T. Kurt, “Serum alcohol is not the same as blood alcohol concentration,” Ann. Emerg. Med. 25(3), 430–431 (1995). 10.1016/s0196-0644(95)70307-1 7864490

[12] M. J. Patterson, S. D. Galloway, and M. A. Nimmo, “Variations in regional sweat composition in normal human males,” Exp. Physiol. 85(6), 869–875 (2000). 10.1111/j.1469-445x.2000.02058.x 11187982

[13] D. Rowett and S. Yaftali, “Serum sodium,” in *Clinical Methods: The History, Physical, and Laboratory Examinations*, 3rd ed. (Butterworths, Boston, 1990), No. 2, Chap. Serum sodium. 21250045

[14] G. Morrison, “Serum chloride,” in *Clinical Methods: The History, Physical, and Laboratory Examinations*, 3rd ed. (Butterworths, Boston, 1990), Chap. Serum chloride, see https://www.ncbi.nlm.nih.gov/books/NBK309/ (Accessed February 7, 2025). 21250045

[15] C.-T. Huang, M.-L. Chen, L.-L. Huang, and I. Mao, “Uric acid and urea in human sweat,” Chin. J. Physiol. 45(3), 109–116 (2002). 12817713

[16] A. Rastegar, “Serum potassium,” in *Clinical Methods: The History, Physical, and Laboratory Examinations*, 3rd ed. (Butterworths, Boston, 1990), No. 4743, Chap. Serum Potassium. 21250045

[17] L. B. Baker and A. S. Wolfe, “Physiological mechanisms determining eccrine sweat composition,” Eur. J. Appl. Physiol. 120, 719–752 (2020). 10.1007/s00421-020-04323-7 32124007 PMC7125257

[18] D. A. Goldstein, “Serum calcium,” in *Clinical Methods: The History, Physical, and Laboratory Examinations*, 3rd ed. (Butterworths, Boston, 1990), No. 5, Chap. Serum calcium, see https://www.ncbi.nlm.nih.gov/books/NBK250/ (Accessed February 7, 2025). 21250045

[19] P. Wacharasint, T.-A. Nakada, J. H. Boyd, J. A. Russell, and K. R. Walley, “Normal-range blood lactate concentration in septic shock is prognostic and predictive,” Shock 38(1), 4–10 (2012). 22552014 10.1097/SHK.0b013e318254d41a

[20] S. W. Brusilow and E. H. Gordes, “Ammonia secretion in sweat,” Am. J. Physiol. Legacy Content 214(3), 513–517 (1968). 10.1152/ajplegacy.1968.214.3.513 5638982

[21] R. J. Barsotti, “Measurement of ammonia in blood,” J. Pediatr. 138(1), S11–S20 (2001). 10.1067/mpd.2001.111832 11148545

[22] J. M. McMillin, “Blood glucose,” in *Clinical Methods: The History, Physical, and Laboratory Examinations*, 3rd ed. (Butterworths, Boston, 1990), Chap. Blood glucose. 21250045

[23] GOV.UK, see https://www.gov.uk/drink-drive-limit for “The drink drive limit” (Accessed: February 7, 2025).

[24] T. Chalklen, Q. Jing, and S. Kar-Narayan, “Biosensors based on mechanical and electrical detection techniques,” Sensors 20(19), 5605 (2020). 10.3390/s20195605 33007906 PMC7584018

[25] L. Wei, Z. Li, Z. Dai, L. Ding, H. Wei, J. Li, H. Zhu, and Y. Zhu, “Wearable sweat management technologies,” Adv. Mater. Technol. 9(7), 2470031 (2024).

[26] S. Majumder, T. Mondal, and M. J. Deen, “Wearable sensors for remote health monitoring,” Sensors 17(1), 130 (2017). 10.3390/s17010130 28085085 PMC5298703

[27] M. Bariya, H. Y. Y. Nyein, and A. Javey, “Wearable sweat sensors,” Nat. Electron. 1(3), 160–171 (2018). 10.1038/s41928-018-0043-y

[28] N. F. A. Sabani, N. Sabani, S. Johari, A. A. Manaf, A. A. Wahab, Z. Zakaria, and A. M. Noor, “A comprehensive review of the recent developments in wearable sweat-sensing devices,” Sensors 22(19), 7670 (2022). 10.3390/s22197670 36236769 PMC9573257

[29] J. Wang, Y. Luo, Z. Zhou, J. Xiao, T. Xu, and X. Zhang, “Epidermal wearable optical sensors for sweat monitoring,” Commun. Mater. 5(1), 1–11 (2024). 10.1038/s43246-024-00518-z

[30] A. Koh, D. Kang, Y. Xue, S. Lee, R. M. Pielak, J. Kim, T. Hwang, S. Min, A. Banks, P. Bastien, M. C. Manco, L. Wang, K. R. Ammann, K.-I. Jang, P. Won, S. Han, R. Ghaffari, U. Paik, M. J. Slepian, G. Balooch, Y. Huang, and J. A. Rogers, “A soft, wearable microfluidic device for the capture, storage, and colorimetric sensing of sweat,” Sci. Transl. Med. 8(366), 366ra165–366ra165 (2016). 10.1126/scitranslmed.aaf2593 PMC5429097 27881826

[31] M. Y. Jia, Q. S. Wu, H. Li, Y. Zhang, Y. F. Guan, and L. Feng, “The calibration of cellphone camera-based colorimetric sensor array and its application in the determination of glucose in urine,” Biosens. Bioelectron. 74, 1029–1037 (2015). 10.1016/j.bios.2015.07.072 26275712

[32] R. Gupta, R. G. Reifenberger, and G. U. Kulkarni, “Cellphone camera imaging of a periodically patterned chip as a potential method for point-of-care diagnostics,” ACS Appl. Mater. Interfaces 6(6), 3923–3929 (2014). 10.1021/am4050426 24564576

[33] S. Patel, R. Jamunkar, D. Sinha, Monisha, T. K. Patle, T. Kant, K. Dewangan, and K. Shrivas, “Recent development in nanomaterials fabricated paper-based colorimetric and fluorescent sensors: A review,” Trends Environ. Anal. Chem. 31, e00136 (2021). 10.1016/j.teac.2021.e00136

[34] Q. Chen, S. Li, X. Tu, and X. Zhang, “Skin-attachable Tb-MOF ratio fluorescent sensor for real-time detection of human sweat pH,” Biosens. Bioelectron. 263, 116606 (2024). 10.1016/j.bios.2024.116606 39089190

[35] K. K. Yeung, T. Huang, Y. Hua, K. Zhang, M. M. F. Yuen, and Z. Gao, “Recent advances in electrochemical sensors for wearable sweat monitoring: A review,” IEEE Sens. J. 21(13), 14522–14539 (2021). 10.1109/JSEN.2021.3074311

[36] A. M. V. Mohan, V. Rajendran, R. K. Mishra, and M. Jayaraman, “Recent advances and perspectives in sweat based wearable electrochemical sensors,” TrAC Trends Anal. Chem. 131, 116024 (2020). 10.1016/j.trac.2020.116024

[37] A. Heller and B. Feldman, “Electrochemical glucose sensors and their applications in diabetes management,” Chem. Rev. 108(7), 2482–2505 (2008). 10.1021/cr068069y 18465900

[38] I. S. Kucherenko, O. O. Soldatkin, S. V. Dzyadevych, and A. P. Soldatkin, “Electrochemical biosensors based on multienzyme systems: Main groups, advantages and limitations—A review,” Anal. Chim. Acta 1111, 114–131 (2020). 10.1016/j.aca.2020.03.034 32312388

[39] A. Gencoglu and A. R. Minerick, “Electrochemical detection techniques in micro- and nanofluidic devices,” Microfluid. Nanofluid. 17(5), 781–807 (2014). 10.1007/s10404-014-1385-z

[40] J. R. Sempionatto, A. A. Khorshed, A. Ahmed, A. N. De Loyola e Silva, A. Barfidokht, L. Yin, K. Y. Goud, M. A. Mohamed, E. Bailey, J. May, C. Aebischer, C. Chatelle, and J. Wang, “Epidermal enzymatic biosensors for sweat vitamin C: Toward personalized nutrition,” ACS Sens. 5(6), 1804–1813 (2020). 10.1021/acssensors.0c00604 32366089

[41] A. Martín, J. Kim, J. F. Kurniawan, J. R. Sempionatto, J. R. Moreto, G. Tang, A. S. Campbell, A. Shin, M. Y. Lee, X. Liu, and J. Wang, “Epidermal microfluidic electrochemical detection system: Enhanced sweat sampling and metabolite detection,” ACS Sens. 2(12), 1860–1868 (2017). 10.1021/acssensors.7b00729 29152973

[42] M. Calderón-Santiago, F. Priego-Capote, B. Jurado-Gámez, and M. D. Luque de Castro, “Optimization study for metabolomics analysis of human sweat by liquid chromatography–tandem mass spectrometry in high resolution mode,” J. Chromatogr. A 1333, 70–78 (2014). 10.1016/j.chroma.2014.01.071 24529403

[43] J. Lee, S. A. Soper, and K. K. Murray, “Microfluidic chips for mass spectrometry-based proteomics,” J. Mass Spectrom. 44(5), 579–593 (2009). 10.1002/jms.1585 19373851

[44] M. Yang, T. Y. Kim, H. C. Hwang, S. K. Yi, and D. H. Kim, “Development of a palm portable mass spectrometer,” J. Am. Soc. Mass Spectrom. 19(10), 1442–1448 (2008). 10.1016/j.jasms.2008.05.011 18565759

[45] J. Min, J. Tu, C. Xu, H. Lukas, S. Shin, Y. Yang, S. A. Solomon, D. Mukasa, and W. Gao, “Skin-interfaced wearable sweat sensors for precision medicine,” Chem. Rev. 123(8), 5049–5138 (2023). 10.1021/acs.chemrev.2c00823 36971504 PMC10406569

[46] A. Childs, B. Mayol, J. A. Lasalde-Ramírez, Y. Song, J. R. Sempionatto, and W. Gao, “Diving into sweat: Advances, challenges, and future directions in wearable sweat sensing,” ACS Nano 18(36), 24605–24616 (2024). 10.1021/acsnano.4c10344 39185844 PMC12129723

[47] T. Wade, S. Malik, L. Ives, N. Ćatić, and S. Kar-Narayan, “Purely electrical detection of electrolyte concentration through microfluidic impedance spectroscopy,” Cell Rep. Phys. Sci. 5(8), 102133 (2024). 10.1016/j.xcrp.2024.102133

[48] S. Robinson, R. T. Maletich, W. S. Robinson, B. B. Rohrer, and A. L. Kunz, “Output of NaCl by sweat glands and kidneys in relation to dehydration and to salt depletion,” J. Appl. Physiol. 8(6), 615–620 (1956). 10.1152/jappl.1956.8.6.615 13331845

[49] D.-H. Choi, A. Thaxton, I. c. Jeong, K. Kim, P. R. Sosnay, G. R. Cutting, and P. C. Searson, “Sweat test for cystic fibrosis: Wearable sweat sensor vs. Standard laboratory test,” J. Cystic Fibrosis 17(4), E35–E38 (2018). 10.1016/j.jcf.2018.03.005 29580829

[50] H. Okawara, T. Sawada, D. Nakashima, Y. Maeda, S. Minoji, T. Morisue, Y. Katsumata, M. Matsumoto, M. Nakamura, and T. Nagura, “Kinetic changes in sweat lactate following fatigue during constant workload exercise,” Physiol. Rep. 10(2), e15169 (2022). 10.14814/phy2.15169 35043587 PMC8767313

[51] M. Certo, A. Llibre, W. Lee, and C. Mauro, “Understanding lactate sensing and signalling,” Trends Endocrinol. Metab. 33, 722–735 (2022). 10.1016/j.tem.2022.07.004 35999109

[52] J. Ouyang, H. Wang, and J. Huang, “The role of lactate in cardiovascular diseases,” Cell Commun. Signaling 21(1), 1–14 (2023). 10.1186/s12964-023-01350-7 PMC10623854 37924124

[53] J. L. Lafuente, S. González, C. Aibar, D. Rivera, E. Avilés, and J. J. Beunza, “Continuous and Non-invasive lactate monitoring techniques in critical care patients,” Biosensors (Basel) 14(3), 148 (2024). 10.3390/bios14030148 38534255 PMC10968200

[54] P. Marty, A. Roquilly, F. Vallée, A. Luzi, F. Ferré, O. Fourcade, K. Asehnoune, and V. Minville, “Lactate clearance for death prediction in severe sepsis or septic shock patients during the first 24 h in intensive care unit: An observational study,” Ann. Intensive Care 3(1), 3 (2013). 10.1186/2110-5820-3-3 23398782 PMC3614435

[55] Z. Li, Y. Wang, Z. Fan, Y. Sun, Y. Sun, Y. Yang, and Z. Zhu, “A dual-function wearable electrochemical sensor for uric acid and glucose sensing in sweat,” Biosensors 13(1), 105 (2023). 10.3390/bios13010105 36671938 PMC9855683

[56] H. A. Petersen, E. N. Miller, P. H. Pham, Kajal, J. L. Katsirubas, H. J. Koltunski, and O. R. Luca, “On the temperature sensitivity of electrochemical reaction thermodynamics,” ACS Phys. Chem. Au 3(3), 241–251 (2023). 10.1021/acsphyschemau.2c00063 37249933 PMC10214520

[57] M. S. Sha, M. R. Maurya, M. Geetha, B. Kumar, A. M. Abdullah, and K. K. Sadasivuni, “A smart colorimetric platform for detection of methanol, ethanol and formic acid,” Sensors 22(2), 618 (2022). 10.3390/s22020618 35062579 PMC8780487

[58] E. B. Neves, J. Vilaca-Alves, N. Antunes, I. M. V. Felisberto, C. Rosa, and V. M. Reis, “Different responses of the skin temperature to physical exercise: Systematic review,” in *Proceedings of the Annual International Conference of the IEEE Engineering in Medicine and Biology Society, EMBS* (IEEE, 2015), Vol. 2015, pp. 1307–1310. 10.1109/EMBC.2015.7318608 26736508

[59] A. Martineikz-Nicolas, M. Meyer, S. Hunkler, J. A. Madrid, M. A. Rol, A. H. Meyer, A. Schötzau, S. Orgül, and K. Kräuchi, “Daytime variation in ambient temperature affects skin temperatures and blood pressure: Ambulatory winter/summer comparison in healthy young women,” Physiol. Behav. 149, 203–211 (2015). 10.1016/j.physbeh.2015.06.014 26072176

[60] J. T. S. Irvine, D. C. Sinclair, and A. R. West, “Electroceramics: Characterization by impedance spectroscopy,” Adv. Mater. 2(3), 132–138 (1990). 10.1002/adma.19900020304

[61] J. Scherbel, P. H. Nguyen, G. Paasch, W. Brütting, and M. Schwoerer, “Temperature dependent broadband impedance spectroscopy on poly-(p-phenylene-vinylene) light-emitting diodes,” J. Appl. Phys. 83(10), 5045–5055 (1998). 10.1063/1.367321

[62] A. Manz, J. Black, K. Pashaev, and D. L. Mills, “Temperature dependence of the low-frequency surface impedance of metals near the extreme anomalous limit,” Phys. Rev. B 17(4), 1721 (1978). 10.1103/PhysRevB.17.1721

[63] M. Zistler, P. Wachter, C. Schreiner, M. Fleischmann, D. Gerhard, P. Wasserscheid, and H. J. Gores, “Temperature dependent impedance analysis of binary ionic liquid electrolytes for dye-sensitized solar cells,” J. Electrochem. Soc. 154(9), B925 (2007). 10.1149/1.2752972

[64] T. Yamaguchi, T. Yonezawa, and S. Koda, “Study on the temperature-dependent coupling among viscosity, conductivity and structural relaxation of ionic liquids,” Phys. Chem. Chem. Phys. 17(29), 19126–19133 (2015). 10.1039/C5CP02335A 26130182

[65] J. Leys, M. Wübbenhorst, C. Preethy Menon, R. Rajesh, J. Thoen, C. Glorieux, and S. Longuemart, “Temperature dependence of the electrical conductivity of imidazolium ionic liquids,” J. Chem. Phys. 128(6), 064509 (2008). 10.1063/1.2827462 18282058

[66] A. Baldwin, E. Yoon, T. Hudson, and E. Meng, “Fluid temperature measurement in aqueous solution via electrochemical impedance,” J. Microelectromech. Syst. 28(6), 1060–1067 (2019). 10.1109/JMEMS.2019.2939811

[67] See https://www.sterlingsensors.co.uk/pt100-resistance-table for “Pt100 Resistance Table” (Accessed November 22, 2024).

[68] S. Feliu, “Electrochemical impedance spectroscopy for the measurement of the corrosion rate of magnesium alloys: Brief review and challenges,” Metals 10(6), 775 (2020). 10.3390/met10060775

[69] M. J. Kadhim and M. I. Gamaj, “Estimation of the diffusion coefficient and hydrodynamic radius (Stokes radius) for inorganic ions in solution depending on molar conductivity as electro-analytical technique—A review,” J. Chem. Rev. 2(3), 182–188 (2020). 10.22034/jcr.2020.106910

[70] A. Bouchoux, H. Roux-de Balmann, and F. Lutin, “Investigation of nanofiltration as a purification step for lactic acid production processes based on conventional and bipolar electrodialysis operations,” Sep. Purif. Technol. 52(2), 266–273 (2006). 10.1016/j.seppur.2006.05.011

[71] See http://ddbonline.ddbst.de/VogelCalculation/VogelCalculationCGI.exe?component=Water for “Liquid dynamic viscosity calculation by Vogel equation (water)” (Accessed November 27, 2024).

[72] L. Rusiniak, “Dielectric properties and structure of water at room temperature. New experimental data in 5 Hz–13 MHz frequency range,” Phys. Chem. Earth Part A 25(2), 201–207 (2000). 10.1016/S1464-1895(00)00032-6

[73] T. Wade and S. Kar-Narayan, “Research data supporting ‘temperature-dependent microfluidic impedance spectroscopy for non-invasive biofluid characterization,’” Apollo – University of Cambridge Repository (2025). 10.17863/CAM.115876 PMC12048175 40322639

